# A Drug Discovery Approach for an Effective Pain Therapy through Selective Inhibition of Nav1.7

**DOI:** 10.3390/ijms23126793

**Published:** 2022-06-18

**Authors:** Gabriele A. Trombetti, Alessandra Mezzelani, Alessandro Orro

**Affiliations:** Institute for Biomedical Technologies, National Research Council (ITB-CNR), 20054 Segrate, Italy; alessandra.mezzelani@itb.cnr.it (A.M.); alessandro.orro@itb.cnr.it (A.O.)

**Keywords:** in silico drug discovery, analgesia, voltage-gated sodium channel, selectivity, paralogs, pore blocking, ADME, chronic pain, monogenic pain disorders

## Abstract

Chronic pain is a widespread disorder affecting millions of people and is insufficiently addressed by current classes of analgesics due to significant long-term or high dosage side effects. A promising approach that was recently proposed involves the systemic inhibition of the voltage-gated sodium channel Nav1.7, capable of cancelling pain perception completely. Notwithstanding numerous attempts, currently no drugs have been approved for the inhibition of Nav1.7. The task is complicated by the difficulty of creating a selective drug for Nav1.7, and avoiding binding to the many human paralogs performing fundamental physiological functions. In our work, we obtained a promising set of ligands with up to 5–40-fold selectivity and reaching 5.2 nanomolar binding affinity by employing a proper treatment of the problem and an innovative differential in silico screening procedure to discriminate for affinity and selectivity against the Nav paralogs. The absorption, distribution, metabolism, and excretion (ADME) properties of our top-scoring ligands were also evaluated, with good to excellent results. Additionally, our study revealed that the top-scoring ligand is a stereoisomer of an already-approved drug. These facts could reduce the time required to bring a new effective and selective Nav1.7 inhibitor to the market.

## 1. Introduction

According to a 2016 estimate, approximately 50 million individuals in the United States suffer from chronic pain, representing 20.4% of the adult population, a figure which likely extends to hundreds of millions globally [1]. Although the majority of these individuals are able to achieve reasonable pain suppression with current analgesics, the long-term side effects are significant. A 2006 study found that 40% of chronic pain sufferers had insufficient pain management and 19% had persistent pain of moderate to severe intensity, which had a significant impact on the patient’s personal life and ability to work [2].

Currently available analgesics include natural and synthetic opioids, paracetamol and non-steroidal anti-inflammatory drugs (NSAIDs), some gabaergic drugs, some steroidal compounds and some antidepressants. However, all these classes of drugs have restrictions on their use due to their metabolism and/or significant side effects. In this context, inhibiting a new biological pathway associated with pain would be extremely beneficial in therapy, with the added benefit of hypothetically allowing for combined approaches, but new receptors representing new targets for drug design and development of molecular compounds are needed. Recently, the utilization of genes that cause rare monogenic disorders as therapeutic targets has sparked considerable interest and is expected to be a promising strategy [3].

In this context, it was observed that individuals carrying a mutation in the voltage-gated sodium channel alpha subunit 9 (SCN9A) gene (ID 603415), which results in a voltage-gated sodium channel (Nav) 1.7. loss-of-function, suffer from congenital insensitivity to pain (CIP) (OMIM #243000), a rare autosomal recessive disorder in which affected individuals are unable to perceive pain from birth to death. CIP insensitivity to pain and comorbid anosmia are the only manifestations of the condition. On the contrary, an excessive Nav1.7 activity, as occurs in erythermalgia (OMIM #133020), results in an acute burning sensation in response to modest heat, further confirming the role of Nav1.7 in pain sensitivity. This makes Nav1.7 an intriguing target for the development of novel analgesic agents and it has been speculated that targeting and inhibiting wild-type Nav1.7 by a ligand should theoretically completely and safely relieve pain while avoiding serious side effects [4,5].

Nav1.7 is a member of the Nav family of large membrane proteins that regulate potential initiation and propagation in excitable cells: under stimulus, these proteins allow Na+ to enter the cell, resulting in depolarization. Structurally, the proteins of this family are composed of a pore-forming α subunit (260 kDa) coupled with one or more β subunits (33–36 kDa) [6]. Nine α subunit isoforms (Nav1.1–1.9) have been identified and functionally characterized [7]; another closely related isoform, Nav2.1 or Nax, is a Na+ channel that has lost its voltage-gated character during evolution, acquiring a concentration-sensitive mechanism [8]. Nav1.7 is expressed specifically in the involuntary nervous system, precisely in nociceptive dorsal root ganglion (DRG) neurons and sympathetic ganglion neurons at the nociceptor nerve endings. Stimulation of these endings induces a transient depolarization of the neuronal membrane that is amplified by the Nav1.7 channel up to a certain threshold, causing the neuron to fire [9]. Recently, it was shown [10] that analgesia caused by Nav1.7 deletion is dependent on the inhibition of neurotransmitter release, and the lack of Nav1.7 does not impair peripheral excitability, while it greatly reduces synaptic transmission from central nociceptors in the spinal cord. This central mechanism is opioid-dependent, can be reversed by central application of opioid antagonists (i.e., naloxone), and involves analgesia only, with anosmia being opioid-independent. For this reason, a synergistic combination of highly specific Nav1.7 antagonists with opioids has been suggested [11,12]. It should be noted, however, that the above mechanism might not be universally accepted as other researchers were able to achieve a virtually complete analgesia by using a Nav1.7 inhibitor compound that has only peripheral effects [13].

The design of a compound that binds the Nav1.7 isoform may be of great value in therapy; however, the structural similarity of the isoforms may result in the cross-binding of non-specific compounds possibly leading to serious side effects. The members of this family that are expressed in the central nervous system appear to regulate a neuronal balance and it was recently postulated that Nav1.1 and Nav1.6 constitute two opposing sides of a neuronal balance between inhibition and activation [14]. In Dravet Syndrome (DRVT) (OMIM #607208), a severe form of drug-resistant epilepsy, the loss of function mutations of the SCN1A gene affect Nav1.1 and lead to a lack of neuronal inhibition in GABAergic interneurons resulting in neuronal hyperactivity. In a preclinical DRVT model, this could be treated either by Nav1.1 selective activators or Nav1.6 selective inhibitors, in order to restore the delicate neuronal balance [14]. In this regard, we anticipate here that the lead compound identified in this work exhibits a higher than 30-fold selectivity against the likelihood of inhibiting Nav1.1, as shown in the Results section. Another crucial and delicate isoform is Nav1.5, the isoform responsible for the initial upstroke of the action potential in cardiac tissue [15,16]. Similarly, significant suppression of this isoform would be unacceptable, and also in this case, we anticipate a high selectivity by the lead compound identified in this work against such a possibility.

Several previous attempts to develop effective analgesic drugs by inhibiting Nav1.7 have been made, but none has been licensed for human use. Some of these are listed below.

- JNJ63955918 is a synthetic version of a tarantula venom peptide that shares portions of its structure. Highly selective for Nav1.7, it has shown profound pain inhibition efficacy at well-tolerated doses in rats [17], and is unique because it binds the closed form of the protein.
- DS-1971a, a powerful and selective Nav1.7 inhibitor characterized by a sulfonamidic moiety, has shown strong effectiveness in animal models (i.e., mice and cynomolgus monkey) of neuropatic pain in preclinical studies [18]. Safety pharmacology and chronic toxicity studies, conducted in vivo by up to 1000 mg/Kg and up to nine months DS-1971a administration, demonstrated that the compound did not induce any adverse effects. Human safety and tolerability [19] and dose study [20] trials were performed but the results have not yet been published.
- Ralfinamide [21] differs from the two compounds above by acting on multiple receptors, including Nav1.7, and is therefore not selective for Nav1.7. Ralfinamide has already successfully completed the phase 2 clinical trial [22]; however, it subsequently failed phase IIb/III trial due to back pain [23]. A phase 3 trial for neuropathic pain is still ongoing [24] and seems promising based on animal studies [23,25].
- An epigenetic approach to Nav1.7 inhibition was attempted [26] by using CRISPR-dCas9 and zinc fingers to epigenetically repress Nav1.7 in animal models, it was possible to obtain long-lasting analgesia, reduce thermal hyperalgesia, and reverse chemotherapy-induced chronic pain, without observable changes in motor function.
- Spider toxin μ-theraphotoxin-Pn3a is a selective inhibitor of Nav1.7 [11] whose analgesic effect was verified by Mueller et al. [12] using a mouse model, both with local and systemic administration. The toxin demonstrated strong anti-allodynic effects in acute post-surgical pain, and showed a superadditive effect with both the opioid oxycodone and the agonist GABA-B Baclofen, while naloxone inhibited its effects, demonstrating involvement of the endogenous opioids in the functioning of μ-theraphotoxin-Pn3a.
- In one paper [13], some benzoxazolinone aryl sulfonamides related to Nav1.7 and selective against Nav1.5 were tested in vitro and in vivo. The in vivo mouse test for the authors’ compound number 17 demonstrated strong and virtually complete inhibition of pain in a formalin paw assay, both for subcutaneous and oral administration, although the dose required to achieve such inhibition appears to be high. Notably, the compound was peripherally restricted and therefore had no effect on the CNS.

These promising earlier works appear to indicate that approaching analgesia through the inhibition of Nav1.7 is indeed possible.

## 2. Results

In order to facilitate a sequential reading of this article, we will briefly summarize here some concepts from the Materials and Methods and Discussion sections that may be useful for interpreting the results. For this research, we applied a novel differential virtual screening procedure to selectively block the Nav1.7 target for analgesia purposes. The virtual screening target was selected as the pore of the Nav channel proteins 1.1, 1.2, 1.4, 1.5, 1.7 with the first four serving as Negative Targets and Nav1.7 as Positive Target for the differential screening. For each ligand, the numerical affinity results obtained by docking against these structures are compared to obtain a measure of selectivity. From the affinity and selectivity measurements, we derive AS_score with a formula that is approximately −affinity×selectivity, a quantity that is used as a ranking in this research work to find ligands that simultaneously have good affinity and good selectivity, giving equal weight to the two features. An ADME analysis is also performed for the top-scoring ligands. Given the complexity of the procedure followed, we refer to the Materials and Methods and Discussion sections for details.

Table 1 and Table 2 report the final results for the virtual screening calculation, showing the best 20 ligands found.

The affinity against the five targets, the best negative target (NT) affinity, the selectivity and the AS_score (see the Discussion section for the definitions), of the best ligands from our computation are listed in Table 1. Table 2 contains the SMILES strings for such compounds.

**Figure 1:**
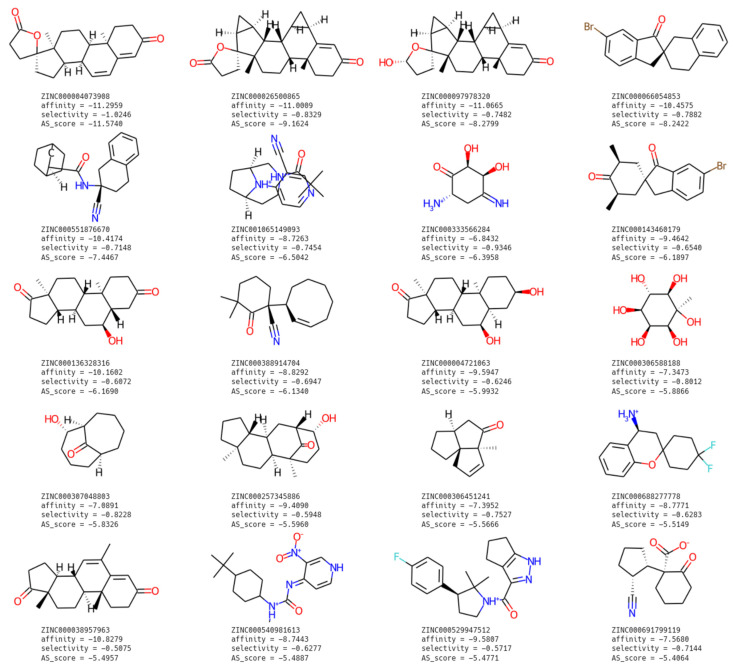
Best molecules at the end of computation.

The 20 top scoring ligands are represented in Figure 1.

After the first few positions, the AS_score of the ligand decreases drastically, and in fact for the ligands beyond the fifth ranked, we expect that a rather advanced ligand optimization work would be required in order to obtain affinity and selectivity scores sufficient for commercialization.

The first four ranked ligands have zero rotatable bonds; from the fifth, i.e., ZINC000551876670, we begin to notice the presence of rotatable bonds (the fifth with two, the sixth with one). However, the group of zero rotatable bonds numerically dominates our 20 best ranked ligands. As expected, the least flexible ligands are among the most selective.

On average, these ligands have 3.5 rings, but they are predominantly non-aromatic (0.45 aromatic rings on average in the best 20 ligands). The number of aromatic rings increases in the first ranked positions reaching about 0.67 aromatic rings per ligand. The average molecular weight is 282.51, but it is higher in the first ligands, reaching an average of 337.18 for the first six ligands. Apart from a few compounds, the majority of them contain nitrogen and/or oxygen atoms essential for H-bond interactions at the binding site. However, hydrophobic interactions appear to predominate over polar interactions in the chemical structure of the selected compounds, thus suggesting the interaction of the ligands with lipophilic pockets inside Nav1.7 protein. Indeed, the narrowest location within the Nav1.7 protein pore, and thus the best location for blocking it, is the terminal part of the pore at the intracellular side, a location where the protein surface is highly hydrophobic, as will be shown later in this chapter.

Most of our lead compounds have good to excellent ADME properties.

**Figure 2:**
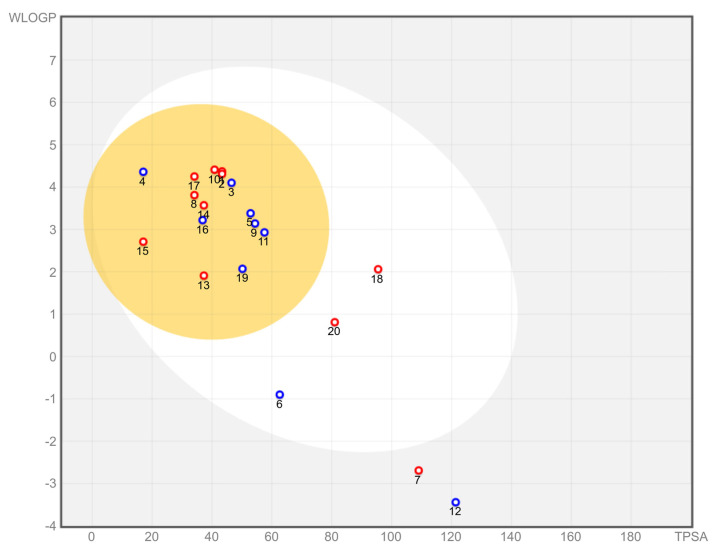
SwissADME BOILED-Egg diagram for brain access and gastrointestinal absorption. The meaning of colors is the following: white region = high intestinal absorption, yellow region = BBB-permeant, red color = not a p-glycoprotein substrate.

Figure 2 depicts the SwissADME BOILED-Egg [27] diagram for our top 20 compounds. The ligands are labeled according to their rank in Table 1 (note that compound 1, scarcely visible, is superimposed to 2 and of the same color). With the exception of compounds 7 and 12, all are predicted to have high intestinal absorption, and with the exception of compounds 6, 7, 12, 18 and 20, all are expected to cross the blood–brain barrier (BBB) and exert their action on the central nervous system (CNS), in addition to peripherally.

Among those that do cross the BBB, compounds 1, 2, 8, 10, 13, 14, 15 and 17 are projected to be non-p-glycoprotein substrates, i.e., unlikely to be eliminated from the CNS via the membrane-bound ATP-driven p-glycoprotein pump, and this should reduce the estimated systemic dose required to obtain therapeutic CNS concentrations.

Table 3 summarizes the main physicochemical properties and predictions by SwissADME. The full SwissADME analysis csv file for our top 20 ligands is available in Supplementary Materials (the SMILES will appear different from those of Table 2, as they are due to being rewritten by the internal SwissADME engine, but are equivalent).

All our top 20 compounds score positively for lead-likeness predictions on Lipinski, Veber, and Egan rules, and all except compounds 4, 6, 7, 12, 13, 14, 15 also score positively for Ghose and Muegge. Regarding lead-likeness by Teague et al. [28], 8 compounds are positively predicted and 12 negatively predicted.

**Figure 3:**
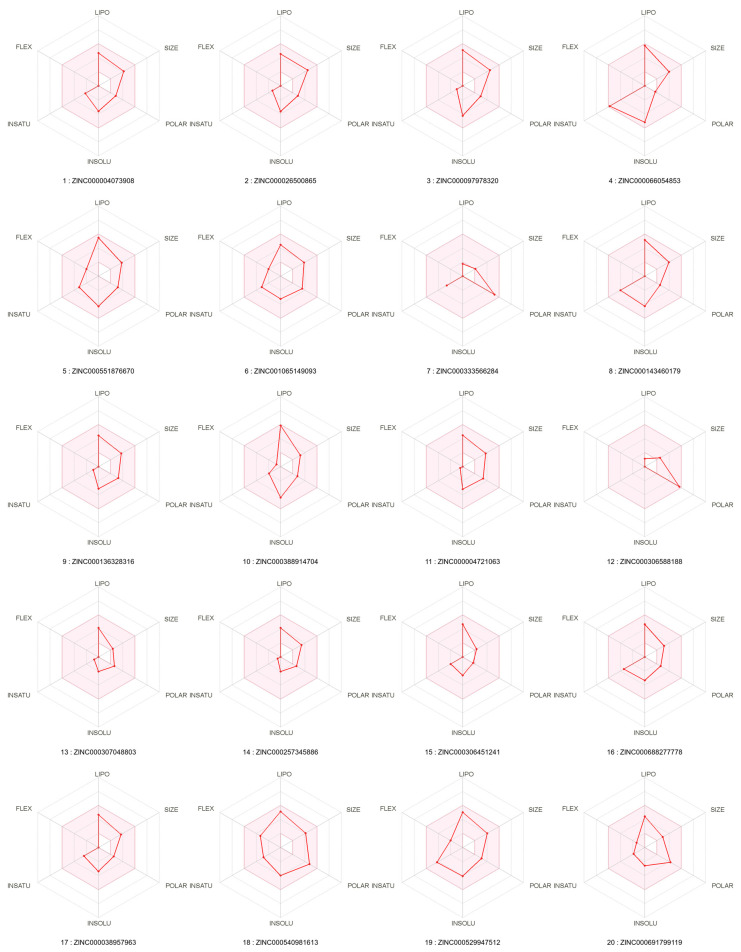
ADME radar plots by SwissADME for oral bioavailability. The pink region represents suitable physicochemical space for oral bioavailability, for each of the six physicochemical parameters and for each of the top 20 ligands presented in this work.

Figure 3 reports the radar plots for oral bioavailability of our top 20 ligands by SwissADME. There is a minimal violation in compounds 4 and 15 for POLAR, and a more significant violation in compounds 7 and 12 for LIPO. All other oral bioavailability parameters are in range for all compounds.

Overall, ADME predictions appear favorable for our top 20 ligands, and are particularly promising for our lead ligand ZINC000004073908, which is the first ligand in the AS_score ranking in Table 1, and which also scores positively for all mentioned SwissADME predictions: high GI absorption, positive BBB crossing, not a p-glycoprotein substrate, all properties in range for oral bioavailability, zero violations in drug-likeness predictions for Lipinski, Veber, Egan, Ghose and Muegge, and positive prediction for Teague et al. lead-likeness.

Our best result is the ligand ZINC000004073908, which has a high affinity against Nav1.7 (ΔG = −11.295949 kcal/mol) and which is a highly selective (ΔΔG = −1.024619 kcal/mol).

The molar activity for ligand ZINC000004073908 at 25 °C can be calculated with the equations and meaning of terms as described in the Materials and Methods. Here, we apply Equations (14)–(16), substituting the following value from Table 1

ΔG(ZINC000004073908) = −11.2959 kcal/mol(1)

resulting in

Kd(ZINC000004073908, 25 °C) ~= 5.248 × 10^−9^ (2)

At 25 °C, the Kd molarity equals 5.248 × 10^−9^; therefore, this is a 5.2 nanomolar compound, officially in the low-micromolar, almost nanomolar range (nanomolar at 25 °C would require ΔG ~= −12.3 kcal/mol).

Its selectivity expressed as the ratio of the Ka constants (selectivity coefficient) can be calculated via (19) and (20), taking the following value from Table 1

sel_ΔΔG(ZINC000004073908) = −1.024619(3)

resulting in

sel_as_Ka_ratio(ZINC000004073908, 25 °C) ~= 5.637(4)

thus, the ligand is at least 5.637 times more affine to Nav1.7 than to any of the other four Nav sodium channel proteins; 5.637 being the worst case, which is for Nav1.4.

The selectivity coefficients against all the four negative target (Nt) protein channels can likewise be evaluated explicitly by (17) and (18), substituting all the needed values from Table 1, we obtain

ΔΔG(ZINC000004073908, Nav1.1) = −2.028139(5)

ΔΔG(ZINC000004073908, Nav1.2) = −1.434114(6)

ΔΔG(ZINC000004073908, Nav1.4) = −1.024619(7)

ΔΔG(ZINC000004073908, Nav1.5) = −2.195519(8)

and then

sel_as_Ka_ratio(ZINC000004073908, Nav1.1, 25 °C) ~= 30.665(9)

sel_as_Ka_ratio(ZINC000004073908, Nav1.2, 25 °C) ~= 11.252(10)

sel_as_Ka_ratio(ZINC000004073908, Nav1.4, 25 °C) ~= 5.637(11)

sel_as_Ka_ratio(ZINC000004073908, Nav1.5, 25 °C) ~= 40.675(12)

Coefficients of selectivity range between 5.637 times and 40.675 times. Specifically, the important Nav1.5 isoform responsible for the initial upstroke of the action potential in cardiac tissue [29,30] is well protected with an over 40-fold difference between the binding ratio and molar activity.

While it is likely that even with these values it would not be possible to skip the ligand optimization phase, we believe these values to be an excellent starting point.

**Figure 4:**
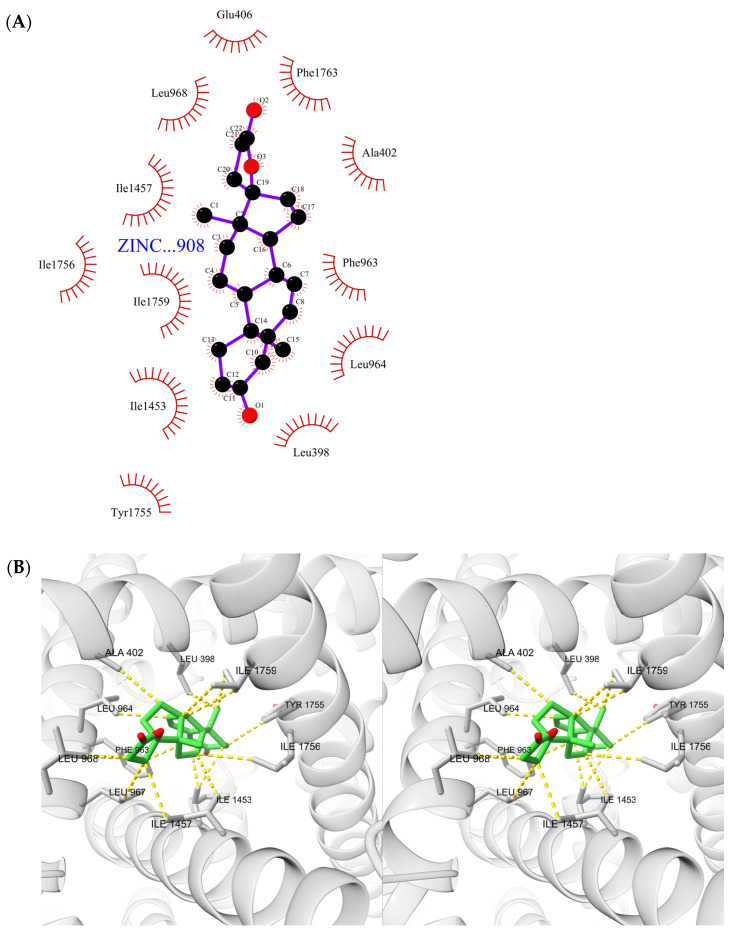
Interactions of first-ranked ligand. (**A**) Interactions as shown in LigPlot+. (**B**) Interactions as detected by PLIP shown in ChimeraX 3D cartoon and stick representation (cross-eye stereoimage).

Figure 4A,B, respectively, show the LigPlot+ [31] depiction of the ligand ZINC000004073908 interactions, and the analogous visualization with ChimeraX [32] (cross-eye stereoimage), in which we mapped the interactions as detected by PLIP [33].

A large amount of hydrophobic interactions are detected by both software programs. Since the hydrophobic interactions result from entropic changes rather than attractive forces between atoms [34], these interactions are substantially broad and do not strictly depend on the position of the side chain at any given instant, and thus they provide the compound with considerable stability. LigPlot+ also detects an additional hydrophobic interaction with Glu406 not detected by PLIP. There are no other types of interaction detected other than hydrophobic, for this molecule.

In the mapping that we have carried out towards ChimeraX of the PLIP output, it is possible to precisely see the 15 nonbonded interactions. The amino acids that it interacts with are: LEU398, ALA402, GLU406 (detected only by LigPlot+), PHE963, LEU964, LEU967, LEU968, ILE1453 (2 times), ILE1457 (2 times), TYR1755, ILE1756, ILE1759 (3 times).

**Figure 5:**
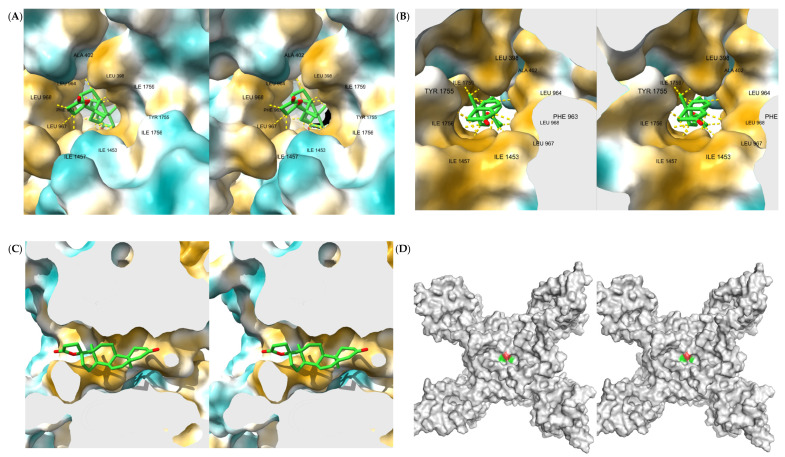
First-ranked ligand in its binding site as seen from outside the pore (**A**), from inside the pore using a section plane in light gray color (**B**), laterally by sectioning the pore using a section plane in light gray color (**C**), and as surface representation to confirm complete blocking of the pore (**D**) (cross-eye stereoimages).

The cross-eye stereoimages shown in Figure 5A,B illustrate the ligand location relative to the protein surface, depicted with hydrophobicity coloring, and the PLIP-detected interactions as shown in Figure 4B. As can be noticed, the terminal section of the pore exhibits a highly hydrophobic surface (gold color), which is leveraged by this ligand to form such a high number of hydrophobic contacts.

In Figure 5C, the positioning of the ligand within the pore is shown from the side via a longitudinal section of the pore: the ligand is substantially facing the exit at the intracellular side, while the rear part of the ligand shows a preference for arranging itself towards the hydrophobic zone, avoiding the limited hydrophilic area available.

Figure 5D was used to assess the complete blockage of the Nav1.7 pore. The figure shows the voltage-gated sodium channel pore-forming alpha subunit with the docked ligand, both rendered in surface mode: the surface smoothly transitions from the protein to the ligand without forming cavities that could indicate imperfect blocking. Color coding: protein in grayscale, ligand with by-atom-type coloring.

## 3. Discussion

### 3.1. Minimization

Minimization has a significant impact on docking.

The presence of gravely incorrect positions of the atoms at the resolutions of our source PDBs (around 3.00 Å) with numerous atomic clashes detected by modeling software, results in unrealistically narrow spaces in some points of the protein surface, preventing ligands from positioning themselves during docking, and excessively wide spaces in other points, allowing poses of the ligand which would not be possible in reality.

In addition to this, proteins are highly flexible structures [35,36]; hence, the presence of any ligand in the coordinates acquisition phase, and therefore in this case of the toxin Tetrodotoxin in 6J8I and of 9Z9 that is (3beta,14beta,17beta,25R)-3-[4-Methoxy-3-(methoxymethyl)butoxy]spirost-5-en in most negative targets, generate short and long range electrostatic changes in proteins, which are not suitable for another ligand, and if the so-altered coordinates were used for a virtual screening skewed energies for the ligands would result, skewed positively or negatively depending on the similarity of the ligand in question with Tetrodotoxin or 9Z9.

All of the above artifacts generate ΔG energy skews that would be particularly nefarious in this work, where we quantitatively measure the ligands’ selectivity through a ΔΔGs energy difference in these ligands against couples of receptors (between Nav1.7 and each of the negative target proteins).

For these reasons, after removing all toxins and other ligands (9SR, NAG, 9Z9, Na+, 6OU) and keeping only the pore-forming alpha subunits, a deep minimization with infinite cutoff was performed so that the overall structure of the five protein pores could be restored.

### 3.2. Docking

All dockings in this work were carried out using practically the same parameters, except for a few cases explained here and in the Materials and Methods section.

As mentioned, our study aimed to find ligands with a high affinity and selectivity against Nav1.7. In terms of affinity (ΔG against Nav1.7), the score used is that of Smina, which is identical to Vina, in the unit of measurement kcal/mol. Regarding the selectivity, our method consists of computing the ΔΔG difference in affinity of a ligand between the ΔG against Nav1.7 and the ΔG against the negative targets. Due to the four negative targets, there are four possibilities for ΔΔG: in this work, we choose the worst case (best Negative Target ΔG, which results in the worst ΔΔG) among the 4 available, for each ligand. The selectivity reported in this work is, hence, an upper bound (a lower bound in absolute value). Note that the selectivity as defined by us has increasingly better values towards the most negative values.

The quantity that is optimized in this work is the following product “−affinity × selectivity”, defined for a ligand L as:

(13) $$\mathrm{AS}\text{\_}\mathrm{score}\left(L\right)=\{\begin{array}{c} \begin{array}{cc} -\mathrm{affinity}\left(\mathrm{Nav}1.7\right)\cdot \mathrm{selectivity}\left(L\right) & \mathrm{if}\left(\mathrm{affinity}\left(\mathrm{Nav}1.7,L\right)<0\right)\wedge \left(\mathrm{selectivity}\left(L\right)<0\right)\\ 0 & \mathrm{otherwise} \end{array} \end{array}$$

For the ligands of interest, AS_score is negative and is maximally negative for the most interesting ligands, similarly to affinity and selectivity. For the cases of the ligands of interest, AS_score is defined to give equal weight to affinity and to selectivity during the ranking operations. For ligands with positive affinity or positive selectivity, AS_score is defined to be zero, effectively excluding such non-interesting ligands from further searches and refinements.

The ligands that had a high ranking obtained at low exhaustiveness, due to the fact that a low exhaustiveness could produce unreliable affinity and selectivity, were refined at a very high exhaustiveness level to prevent the presence of false positives among the first ranked.

Regarding the choice of a different size of the docking boxes for the positive target Nav1.7 as compared to the four negative targets (Nav1.1, Nav1.2, Nav1.4, Nav1.5), this depends on the conservative approach that has been used in our work. It was not possible to extend the docking box against the positive target up to 20 Å laterally, otherwise the great majority of the best poses for all ligands would have been detected about midway into the pore, where the sequences are less conserved, but, unfortunately, in that position, the Nav proteins pores are very wide and a reasonably sized ligand fails to block the pore completely. The other possibility for making the two boxes equal was to restrict the docking box against the negative targets to 7.5 Å laterally as it is for the positive target: in this case, we would have risked obtaining falsely good results. This is due to the fact that the docking software could have detected poor energies for a ligand against the negative targets potentially because some atoms could not exactly fit into the narrow box in the case of the negative targets; in this case, a good negative pose would have been discarded, even though such a pose could acceptably block the pore, and a falsely high selectivity (falsely good result) would have been registered. This would have required us to manually validate a large amount of the top results to verify that those were not falsely good. Our conservative approach, as described here, can instead potentially discard good results (falsely bad ligands), but does not allow falsely good results to be produced. Apart from the above reason for using two different box sizes, the docking boxes chosen are virtually the largest boxes that could meaningfully be created in order to target the pore.

### 3.3. ADME Analysis

We believe the SwissADME radar plots can benefit from an additional explanation beyond the one supplied by [37].

The pink region in the radar plots is the area for expected high oral bioavailability of the molecule, and is declared to be plotted according to the following rules (taken from [38,39] as originally cited in [37]):

- LIPO(Lipophilicity): −0.7 < XLOGP3 < +5.0;
- SIZE(Mol.Weight): 150 g/mol < MW < 500 g/mol;
- POLAR(Polarity): 20 Å^2^ < TPSA < 130 Å^2^;
- INSOLU(Insolubility): log_S > −6;
- INSATU(Insaturation): Csp3 fraction > 0.25;
- FLEX(Flexibility): num. rotatable bonds < 9.

However, the INSATU spoke as displayed in the plot appears to be the reciprocal of the declared formula, 1/Csp3_fraction, so to transform the greater-than inequation in a less-than inequation; this results in the pink region for INSATU being relocated towards the center of the plot. Similarly, INSOLU plotting appears to have the signs flipped as −log_S < 6 so to bring the pink region to the inside again.

Regarding the LIPO spoke, the depiction of the pink region is ambiguous because it is highly discontinuous between the clockwise side of LIPO and the counterclockwise side of LIPO, the counterclockwise side being much more tolerant to small values of LIPO than the clockwise side of it. The correct reference pink region for LIPO is the one at the clockwise side of LIPO (the strictest one). The pink region is again discontinuous and hence ambiguous at POLAR; in this case, the clockwise side is tolerant to small values. In the case of POLAR, the correct pink region to be used for reference is the one at the counterclockwise side of POLAR (again the strictest one).

### 3.4. Further Insights on Methodology and Results

A further discussion of some intermediate or final results of the calculation is presented below.

**Figure 6:**
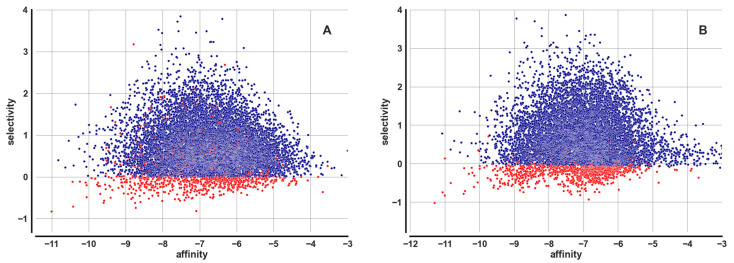
Affinity vs. selectivity, scatterplot. (**A**) before the similarity search. (**B**) after the similarity search.

Figure 6A,B are scatterplots of affinity (against Nav1.7, implied) versus selectivity. The best ligands are located at the lower left. Figure 6A (left) shows the scatterplot for the dockings before the similarity search, while 6B shows it after the similarity search. Red color represents refined values, which are always present for ligands with negative selectivity and occasionally present for some ligands with positive selectivity. There are two reasons for the presence of red dots in the positive selectivity zone: (1) because following refinement, some ligands that had negative selectivity were recalculated as having positive selectivity (due to new good poses found for the negative targets), and (2) because the ligands in the negative selectivity zone were less than 1000; hence, our algorithm selected additional points to refine, which were randomly located since AS_score is non-ordered in the positive selectivity zone. Such points mostly remain in the positive selectivity zone after refinement.

In pre-similarity docking, the presence of some interesting ligands is already apparent, most notably a ligand with affinity approximately −11 (in fact −11.000865 is the value from Table 1) and selectivity approximately −0.8 (in fact −0.832883). This ligand is ZINC000026500865 which has AS_score −9.162432 ranked #2 in the final scoring.

The Tanimoto similarity search manages to interpose the ligands ranked #1 (ZINC000004073908), #3 (ZINC000097978320) and #4 (ZINC000066054853) in an already good ranking before #5 (ZINC000551876670) which was already present pre-similarity with ranking #2.

The majority of the ligands concentrate at affinity around −7 and positive selectivity. Average positive selectivity was expected and derives from the fact of being unfavorably unbalanced, as the selectivity formula is a min( ) of the affinities against the four negative targets, selecting the best affinity against any negative target (four negative targets), which, on average, is a better affinity than that against the positive target (one positive target). Indeed, the median for the selectivity is 0.574040, while the two quartiles are 0.186466 and 1.121636, respectively, showing a clear bias towards positive values.

**Figure 7:**
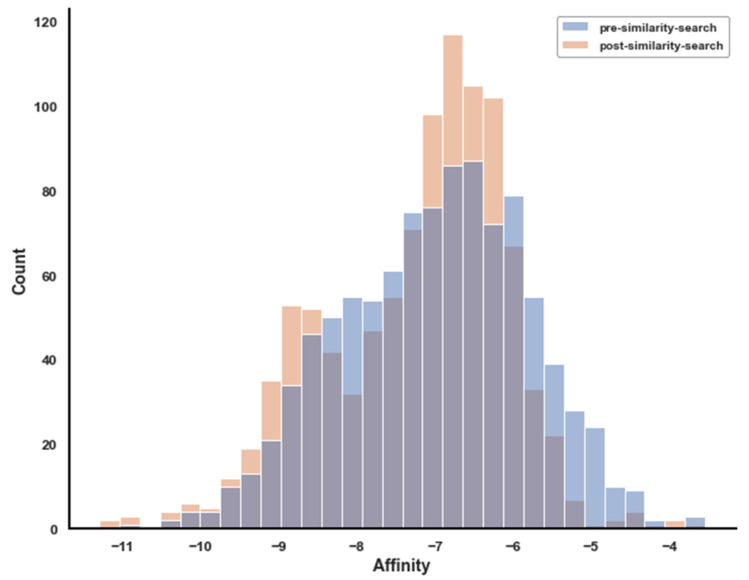
Positive target affinity change post- vs. pre-similarity search, histogram. Only refined points are shown, those that correspond to the top 1000 ligands as ranked by AS_score.

The histogram in Figure 7 approximately displays the improvement in affinity against the positive target Nav1.7 occurring before and after the similarity search; this essentially indicates the effectiveness of the similarity-based selection of the ligands over the random selection used in the earlier dockings.

It can be noted that low affinity ligands improve across the similarity search better than high affinity ligands, and that there is a peculiar aggregation towards an affinity of −6.8, which, however, is a value too low to be of interest. Among the best energies, there is a distinct improvement at affinity around −11, which is what allowed us to discover significantly better lead ligands by leveraging the similarity search compared to what was possible without it.

**Figure 8:**
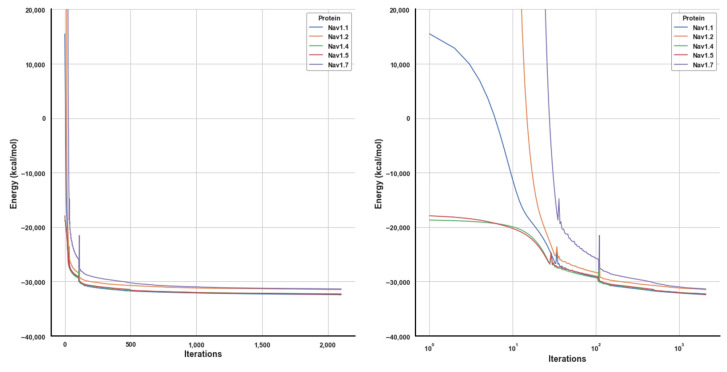
Minimization energy. Left: linear *x* axis. Right: logarithmic *x* axis.

Figure 8 shows the forcefield energy during receptors minimization: the energy decreases rapidly in the initial minimization cycles, but beyond 2000 conjugate gradient cycles, obtaining further appreciable improvements would have required an enormous computational effort.

The energy of the five proteins appears to converge towards two distinct asymptotes: the first cluster has final energy at: Nav1.7 = −3.1365 × 10^4^ and Nav1.2 = −3.1473 × 10^4^; while the other cluster is at: Nav1.1 = −3.2433 × 10^4^, Nav1.4 = −3.2274 × 10^4^ and Nav1.5 = −3.2329 × 10^4^ (kcal/mol). We are currently unsure on how to explain this spontaneous clustering, as the amino acid sequence in the 5 proteins differs significantly.

### 3.5. Patentability

We performed a patent search for our lead ligand ZINC000004073908.

According to the Patentscope [40] search engine by WIPO [41], the lead molecule presented in this work ZINC000004073908 matches 10 patents when chirality information is omitted, while it results in zero patent matches when full chirality information is specified. Indeed, the matches appear to be intended for Canrenone, a stereoisomer of ZINC000004073908, a steroidal antimineralocorticoid drug related to spironolactone currently marketed as a diuretic. Canrenone itself with complete chirality information matches 59 patents.

The fact that ZINC000004073908 is a stereoisomer of a marketed drug indeed suggests that its tolerability in humans is likely (though not certain) to be high, its toxicity likely to be low, the half-life acceptable, and it is expected to have a good bioavailability, similar to that of Canrenone. These facts could significantly shorten the research and experimentation for this Nav1.7 channel blocker.

On the downside, it would be necessary to ensure that patents targeting Canrenone without chirality information and possibly related molecules are circumvented during the ligand optimization phase.

The canonical SMILES for ZINC000004073908 and for Canrenone are, respectively, the following:

C[C@]12CC[C@@H]3[C@@H](C=CC4=CC(=O)CC[C@@]43C)[C@H]1CC[C@@]21CCC(=O)O1

C[C@]12CC[C@H]3[C@@H](C=CC4=CC(=O)CC[C@@]43C)[C@@H]1CC[C@@]21CCC(=O)O1

after removing the chirality information, they both become

CC12CCC(=O)C=C1C=CC1C2CCC2(C)C1CCC21CCC(=O)O1

**Figure 9:**
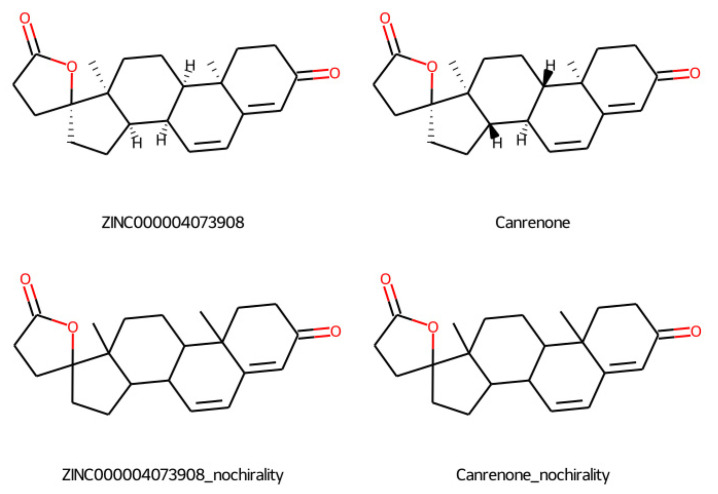
Our lead ZINC000004073908 and Canrenone side to side, with and without chirality information.

Figure 9 shows a 2D depiction of ZINC000004073908 and Canrenone side-to-side, with and without chirality information.

We also tested Canrenone by docking it against Nav1.7, but the affinity of Canrenone resulted to be much lower than that of its stereoisomer ZINC000004073908. The affinity of Canrenone was computed as −8.868431 kcal/mol, i.e., 2.427518 kcal/mol worse than for ZINC000004073908, which implies a required molar concentration 60.171 times higher than that required by our lead, making it impractical for any use against Nav1.7. Furthermore, Canrenone is not selective for Nav1.7.

## 4. Materials and Methods

### 4.1. Targets and Coordinate Files

For the positive target, we used the 6J8I PDB coordinates from RCSB by Shen H. et al. [42], showing the protein voltage gated sodium channel Nav1.7 in complex with Tetrodotoxin and ProTx-II, year 2019, version 2, resolution 3.20 Å.

The following RCSB coordinate files were used as negative targets (NT):

- 7DTD for Nav1.1: year 2021, resolution 3.30 Å, version 1;
- 6J8E for Nav1.2: year 2019, resolution 3.00 Å, version 2;
- 6AGF for Nav1.4: year 2018, resolution 3.20 Å, version 2;
- 7DTC for Nav1.5: year 2021, resolution 3.30 Å, version 1;

of which the PDB versions were used. All files consist of Cryo-EM coordinates.

For our work, all ligands were removed from the five proteins and only the pore-forming alpha subunit was retained in each.

### 4.2. Minimization

For the minimization phase, the five proteins were prepared for the Amber [43] simulator, in implicit solvent igb = 5 with pbradii mbondi2 [44], forcefield ff14SB [45]. The calculation of the energy contribution by the solvent accessible surface was activated.

With these settings, a deep minimization was performed on the CPU with the 100 steepest descent cycles followed by 2000 cycles of conjugate gradient minimization with infinite cutoff.

The minimized versions of the five proteins were used for the docking as described below.

### 4.3. Ligand Dataset

Six hundred thousand (600 k) ligands equally distributed between zero, one and two rotatable bonds were downloaded from the Zinc database [46] by means of a direct https query. The ligands were selected exclusively from the “for sale” set in order to ensure that the leads found were synthesizable and available for purchase and were not molecules of unknown synthesizability, theoretical, or possibly of natural origin.

We confirmed that by downloading the ligands with a direct https query, the ligands were fetched in a random order from the pool of ligands present in the online Zinc database.

The 600 k ligands thus downloaded were used in the subsequent phases as described below.

### 4.4. Docking Runs

The virtual screening was accomplished by means of a series of docking runs performing incremental searches and incremental refinements from the available ligand pool. The software used for all docking runs is Smina [47], a derivative of Vina [48]. We confirmed that Smina provided identical poses and energies to Vina, at least in the case of a launch with high exhaustiveness.

Firstly, a random set of 10 k ligands was selected for an initial docking run from the 600 k ligands pool described above, i.e., 1/60 of the total available.

The docking box was created differently for the positive target than for the negative targets. For the positive target, Nav1.7, a cuboid of 7.5 × 7.5 × 44 Å was created, while for the negative targets, the cuboid was 20 × 20 × 44 Å. In both cases, it was aligned as centered on the pore and slightly protruding at both ends. Regarding the reasons for which we had to choose different docking box sizes for the positive and negative targets, see the Discussion section.

The exhaustiveness was set to 4 (low).

The best 1000 ligands from the above docking runs as ranked by AS_score (−affinity × selectivity, see Discussion section) were selected for a refinement run, where they were docked with exhaustiveness set to 32, but otherwise similarly to before. An exhaustiveness of 32 is among the highest used in the literature.

The top 40 ligands from the above refinement run were chosen as seeds for the Tanimoto-based similarity search as described below, which produced a set of 10 k ligands which were further docked.

The new set of 10 k similarity-based ligands was docked similarly to the above, with exhaustiveness set to 4 (low).

Finally, the best 1000 ligands from the above docking run, ranked by AS_score, were again selected for a refinement docking run using a high exhaustiveness, set to 32. This formed the final result for our computation, again ranked by AS_score.

### 4.5. Similarity Search

The 40 seed ligands generated from the early docking runs, as described above, were used to conduct a similarity search across the 600 k ligands of the pool. For each of the seed ligands, 250 ligands in decreasing order of similarity were selected, totaling 10,000 ligands. The similarity score was calculated using rdkit [49] fingerprints and Tanimoto similarity.

For each seed ligand, 250 ligands were selected based on the similarity score, according to a distribution that is denser to the side of 1.0 (most similar) but reaches down to 0.0 (least similar). The distances between the selected points were derived from a geometric progression. In doing this, the seed ligand was forcibly selected for slot number 1, ensuring that the output from the similarity search included the 40 best ligands from the input.

The similarity search output was used for further dockings as described above.

### 4.6. ADME Analysis

The absorption, distribution, metabolism, and excretion (ADME) analysis was performed using the widely known SwissADME [37] service. The input consisted of the SMILES strings corresponding to the top 20 compounds identified during the docking process, ordered by AS_score.

### 4.7. Kd and Selectivity Calculations

For a ligand L, once the ΔG(L) affinity value against the positive target Nav1.7 is known, the following formula can be used to calculate the dissociation constant Kd

Kd(L, T) = exp(ΔG(L)/(R*T))(14)

where

R = 0.0019872 kcal/(K*mol)        (gas constant)(15)

T = 298.15 K               (25 °C)(16)

The target of the ligand in this work is implicitly assumed to be the positive target Nav1.7 when not specified.

The selectivity of a compound towards Nav1.7 compared to a negative target Nt can be expressed as the ratio of the Ka constants (selectivity coefficients), once the required ΔG affinities are known

sel_as_Ka_ratio(L, Nt, T) = exp(ΔΔG(L, Nt)/(−R*T))(17)

ΔΔG(L, Nt) = ΔG(L, Nav1.7) − ΔG(L, Nt)(18)

In this work, we refer to “selectivity” of a ligand L without specifying either the positive target or the negative target when the positive target is Nav1.7 and the negative target is the most affine of Nav1.1, Nav1.2, Nav1.4, and Nav1.5. In this case, the above formula is written as:

sel_as_Ka_ratio(L, T) = exp(sel_ΔΔG(L)/(−R*T))(19)

where

sel_ΔΔG(L) = ΔG(L, Nav1.7) − min{ΔG(L,Nt) : Nt ∈ {Nav1.1, Nav1.2, Nav1.4, Nav1.5}}(20)

## 5. Conclusions

In this work, we have shown how it is possible to obtain leads of considerable affinity and selectivity that can be used for pain therapy by exploiting a different pathway from those used by current analgesic drugs. This could result in drugs that may be combined with or replace currently available analgesics, allowing for increased combined dosage at a tolerable amount of side effects.

Despite the close similarity of paralogous proteins found in humans and forming unwanted targets, our work demonstrates good success for affinity and selectivity against our target protein channel Nav1.7, finding a set of high-scoring ligands.

In addition, this work evaluated the ADME characteristics for the top-scoring ligands presented, showing on average very good scores for predicted oral availability, GI absorption, BBB crossing, non-substrate for p-glycoprotein and drug-likeness, and fair results for lead-likeness. Our lead ligand, in particular, achieves very high scores in ADME and predicts positively for all mentioned characteristics simultaneously.

Interestingly, our lead ligand happens to be a stereoisomer of a marketed drug, and although we have verified that the marketed drug in question is not usable in place of our lead, the similarity between the two molecules is a further indication that our lead is expected to have good bioavailability and low toxicity.

These findings should hopefully reduce the time required to market a novel analgesic drug based on Nav1.7 inhibition.

## Figures and Tables

**Table 1:** Final virtual screening results. Measurement units for columns 3 to 9 are kcal/mol, last column is in kcal^2^/mol^2^. Columns 3–6 report the affinity values of the compounds against the Negative Targets Nav1.1, Nav1.2, Nav1.4, Nav1.5; Column 8 is the affinity value of the compounds against the Positive Target Nav1.7; Column 7 is the best value from columns 3–6; Column 9 is the difference between Column 8 and Column 7; Column 10 is the AS_score approximately equal to −col8×col9 (see Discussion section for the proper definition). Ranking is by AS_score.

	Rank	Nav1.1aff	Nav1.2aff	Nav1.4aff	Nav1.5aff	Best_NT_aff	Nav1.7aff	Selectivity	AS_Score
ZINC000004073908	1	−9.267810	−9.861835	−10.271330	−9.100430	−10.271330	−11.295949	−1.024619	−11.574045
ZINC000026500865	2	−10.124042	−10.160164	−10.167982	−9.642671	−10.167982	−11.000865	−0.832883	−9.162432
ZINC000097978320	3	−10.151968	−10.040705	−10.318269	−9.648543	−10.318269	−11.066463	−0.748195	−8.279869
ZINC000066054853	4	−9.406034	−9.650142	−9.669319	−8.468196	−9.669319	−10.457479	−0.788159	−8.242159
ZINC000551876670	5	−9.570515	−9.702531	−9.458369	−8.799217	−9.702531	−10.417365	−0.714834	−7.446689
ZINC001065149093	6	−7.405717	−7.432326	−7.980913	−7.507424	−7.980913	−8.726273	−0.745359	−6.504209
ZINC000333566284	7	−5.905749	−5.838212	−5.908603	−5.783346	−5.908603	−6.843217	−0.934615	−6.395771
ZINC000143460179	8	−8.608278	−8.117563	−8.810167	−8.102057	−8.810167	−9.464184	−0.654016	−6.189732
ZINC000136328316	9	−8.542331	−9.541473	−9.553011	−9.110066	−9.553011	−10.160188	−0.607177	−6.169030
ZINC000388914704	10	−8.134428	−7.945611	−7.901132	−7.809019	−8.134428	−8.829168	−0.694740	−6.133979
ZINC000004721063	11	−8.705332	−8.725003	−8.970100	−8.721251	−8.970100	−9.594739	−0.624639	−5.993244
ZINC000306588188	12	−6.317635	−6.545392	−6.513891	−6.546165	−6.546165	−7.347348	−0.801182	−5.886565
ZINC000307048803	13	−6.024336	−6.224265	−6.000527	−6.266380	−6.266380	−7.089138	−0.822757	−5.832639
ZINC000257345886	14	−8.598330	−8.502018	−8.803064	−8.814258	−8.814258	−9.409009	−0.594751	−5.596021
ZINC000306451241	15	−6.462030	−6.415341	−6.642433	−6.582372	−6.642433	−7.395166	−0.752733	−5.566587
ZINC000688277778	16	−7.826337	−8.124866	−8.148812	−8.006941	−8.148812	−8.777143	−0.628330	−5.514944
ZINC000038957963	17	−9.130526	−10.320339	−9.946248	−9.225662	−10.320339	−10.827887	−0.507547	−5.495666
ZINC000540981613	18	−7.828243	−7.830225	−8.068832	−8.116579	−8.116579	−8.744275	−0.627696	−5.488747
ZINC000529947512	19	−8.800838	−9.008982	−8.924126	−8.730454	−9.008982	−9.580667	−0.571685	−5.477122
ZINC000691799119	20	−6.776092	−6.853613	−6.591362	−6.814666	−6.853613	−7.567987	−0.714374	−5.406370

**Table 2:** SMILES for the compounds in Table 1.

	Rank	SMILES
ZINC000004073908	1	C[C@]12CC[C@@H]3[C@@H](C=CC4=CC(=O)CC[C@@]43C)[C@H]1CC[C@@]21CCC(=O)O1
ZINC000026500865	2	C[C@]12CC[C@@H]3[C@@H]([C@H]4C[C@H]4C4=CC(=O)CC[C@@]43C)[C@H]1[C@@H]1C[C@@H]1[C@@]21CCC(=O)O1
ZINC000097978320	3	C[C@]12CC[C@@H]3[C@@H]([C@H]4C[C@H]4C4=CC(=O)CC[C@@]43C)[C@H]1[C@@H]1C[C@@H]1[C@@]21CC[C@H](O)O1
ZINC000066054853	4	O=C1c2cc(Br)ccc2C[C@@]12CCc1ccccc1C2
ZINC000551876670	5	N#C[C@]1(NC(=O)[C@@H]2CC3CCC2CC3)CCc2ccccc2C1
ZINC001065149093	6	CC(C)(C)C(=O)[NH+]1CC[C@H]2CC[C@@H](C1)[NH+]2c1ccncc1C#N
ZINC000333566284	7	N=C1C[C@H]([NH3+])C(=O)[C@@H](O)[C@H]1O
ZINC000143460179	8	C[C@H]1C[C@]2(Cc3ccc(Br)cc3C2=O)C[C@@H](C)C1=O
ZINC000136328316	9	C[C@]12CC[C@H]3[C@@H](C[C@H](O)[C@H]4CC(=O)CC[C@@]43C)[C@@H]1CCC2=O
ZINC000388914704	10	CC1(C)CCC[C@](C#N)([C@@H]2C=CCCCCC2)C1=O
ZINC000004721063	11	C[C@]12CC[C@H]3[C@@H](C[C@H](O)[C@@H]4C[C@H](O)CC[C@]34C)[C@@H]1CCC2=O
ZINC000306588188	12	C[C@]1(O)[C@@H](O)[C@@H](O)[C@@H](O)[C@H](O)[C@H]1O
ZINC000307048803	13	O=C1[C@H]2CCCC[C@@H]1[C@@H](O)CCC2
ZINC000257345886	14	C[C@]12CC[C@@H](O)[C@@H](C[C@H]3[C@@H]1CC[C@@]1(C)CCC[C@H]31)C2=O
ZINC000306451241	15	C[C@]12C=CC[C@]13CCC[C@H]3CC2=O
ZINC000688277778	16	[NH3+][C@H]1CC2(CCC(F)(F)CC2)Oc2ccccc21
ZINC000038957963	17	CC1=C[C@H]2[C@@H]3CCC(=O)[C@@]3(C)CC[C@@H]2[C@@]2(C)CCC(=O)C=C12
ZINC000540981613	18	C[NH+](C(=O)N=c1cc[nH]cc1[N+](=O)[O-])C1CCC(C(C)(C)C)CC1
ZINC000529947512	19	CC1(C)[C@H](c2ccc(F)cc2)CC[NH+]1C(=O)c1n[nH]c2c1CCC2
ZINC000691799119	20	N#C[C@@H]1CCC[C@@H]1[C@]1(C(=O)[O-])CCCCC1=O

**Table 3:** Physicochemical properties as computed by SwissADME. Most columns are unmodified SwissADME columns, and in particular the BBB-permeant and Pgp (p-glycoprotein) substrate values are the same values as can be inspected from the BOILED-egg diagram. The “Drug-likeness Score” in this table is computed as the number of predictors that score positively for a certain molecule among: Lipinski, Ghose, Veber, Egan and Muegge. The “Cytochrome inhibitions” represents the number of Cytochrome P450 enzymes expected to be inhibited among CYP1A2, CYP2C19, CYP2C9, CYP2D6 and CYP3A4.

Molecule	MW	Csp3 Fraction	MR	TPSA	XLOGP3	ESOL Log S	GI Absorption	BBB Permeant	Pgp Substrate	Cyto- Chrome Inhibitions	log Kp (cm/s)	Drug-Likeness Score	Bio- Availability Score	Lead-Likeness #Violations	Synthetic Accessibility
1	340.46	0.73	97.35	43.37	2.68	−3.64	High	Yes	No	1	−6.47	5	0.55	0	5.46
2	366.49	0.83	103.21	43.37	2.47	−3.67	High	Yes	No	0	−6.78	5	0.55	1	5.63
3	368.51	0.88	104.17	46.53	3.40	−4.27	High	Yes	Yes	0	−6.13	5	0.55	1	6.04
4	327.22	0.28	83.88	17.07	4.56	−5.19	High	Yes	Yes	3	−5.06	4	0.55	1	3.41
5	308.42	0.60	90.50	52.89	4.09	−4.32	High	Yes	Yes	2	−5.28	5	0.55	1	4.18
6	314.43	0.61	98.61	62.63	2.34	−3.26	High	No	Yes	0	−6.56	4	0.55	0	4.17
7	159.16	0.67	38.92	109.02	−2.46	0.72	Low	No	No	0	−9.02	3	0.55	1	3.00
8	321.21	0.50	78.66	34.14	3.52	−4.28	High	Yes	No	3	−5.76	5	0.55	1	3.86
9	304.42	0.89	86.03	54.37	2.26	−3.15	High	Yes	Yes	0	−6.55	5	0.55	0	4.00
10	259.39	0.76	78.56	40.86	4.79	−4.40	High	Yes	No	1	−4.48	5	0.55	1	3.92
11	306.44	0.95	86.99	57.53	2.35	−3.22	High	Yes	Yes	0	−6.50	5	0.55	0	4.19
12	194.18	1.00	40.66	121.38	−3.52	1.17	Low	No	Yes	0	−9.98	3	0.55	1	3.69
13	182.26	0.91	52.12	37.30	1.77	−2.09	High	Yes	No	0	−6.16	4	0.55	1	3.42
14	276.41	0.94	81.03	37.30	1.77	−2.09	High	Yes	No	0	−6.16	4	0.55	1	3.42
15	176.25	0.75	52.66	17.07	2.70	−2.63	High	Yes	No	1	−5.46	4	0.55	1	4.57
16	254.30	0.57	66.66	36.87	2.67	−3.35	High	Yes	Yes	0	−5.96	5	0.55	0	3.41
17	298.42	0.70	88.73	34.14	2.71	−3.40	High	Yes	No	1	−6.20	5	0.55	0	5.02
18	335.42	0.65	96.29	95.48	3.50	−3.98	High	No	No	1	−5.86	5	0.55	0	4.13
19	328.40	0.47	95.58	50.19	3.29	−4.09	High	Yes	Yes	0	−5.97	5	0.55	0	3.38
20	234.27	0.77	59.90	80.99	2.30	−2.61	High	No	No	0	−6.10	5	0.56	1	3.08

## Data Availability

Data is available upon request from the corresponding authors.

## Supplementary Materials

The following supporting information can be downloaded at: https://www.mdpi.com/article/10.3390/ijms23126793/s1.
